# Hypoxia in Bone and Oxygen Releasing Biomaterials in Fracture Treatments Using Mesenchymal Stem Cell Therapy: A Review

**DOI:** 10.3389/fcell.2021.634131

**Published:** 2021-08-20

**Authors:** Seoh Wei Teh, Avin Ee-Hwan Koh, Jia Bei Tong, Xiaoyun Wu, Antony V. Samrot, Sanjiv Rampal, Pooi Ling Mok, Suresh Kumar Subbiah

**Affiliations:** ^1^Department of Medical Microbiology, Faculty of Medicine and Health Sciences, Universiti Putra Malaysia, Serdang, Malaysia; ^2^Department of Biomedical Sciences, Faculty of Medicine and Health Sciences, Universiti Putra Malaysia, Serdang, Malaysia; ^3^Department of Technology, Research Center for Hua-Da Precision Medicine of Inner Mongolia Autonomous Region, Hohhot, China; ^4^School of Bioscience, Faculty of Medicine, Bioscience and Nursing, MAHSA University, Jenjarom, Malaysia; ^5^Department of Orthopedics, Faculty of Medicine and Health Sciences, Universiti Putra Malaysia, Serdang, Malaysia; ^6^Center for Materials Engineering and Regenerative Medicine, Bharath Institute of Higher Education and Research, Bharath University, Chennai, India

**Keywords:** bone ischemia, bone fracture, hypoxia, stem cells, oxygen-releasing biomaterials

## Abstract

Bone fractures have a high degree of severity. This is usually a result of the physical trauma of diseases that affect bone tissues, such as osteoporosis. Due to its highly vascular nature, the bone is in a constant state of remodeling. Although those of younger ages possess bones with high regenerative potential, the impact of a disrupted vasculature can severely affect the recovery process and cause osteonecrosis. This is commonly seen in the neck of femur, scaphoid, and talus bone. In recent years, mesenchymal stem cell (MSC) therapy has been used to aid in the regeneration of afflicted bone. However, the cut-off in blood supply due to bone fractures can lead to hypoxia-induced changes in engrafted MSCs. Researchers have designed several oxygen-generating biomaterials and yielded varying degrees of success in enhancing tissue salvage and preserving cellular metabolism under ischemia. These can be utilized to further improve stem cell therapy for bone repair. In this review, we touch on the pathophysiology of these bone fractures and review the application of oxygen-generating biomaterials to further enhance MSC-mediated repair of fractures in the three aforementioned parts of the bone.

## Bone Ischemia

**FIGURE 1:**
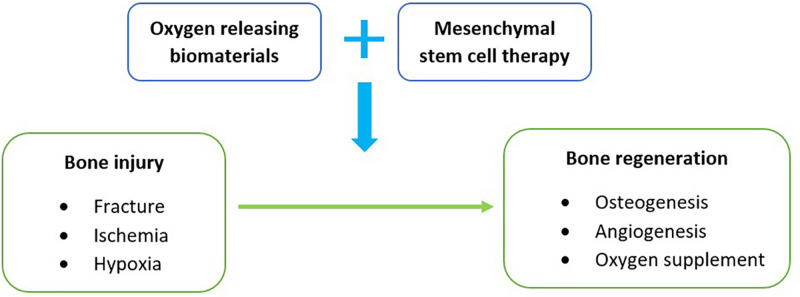
A schematic diagram of the review summary.

This review is mainly focused on the use of oxygen releasing biomaterials with mesenchymal stem cell therapy to address hypoxia in the treatment of bone fractures (Figure 1). Bone fracture cases are commonly presented in varying degrees of severity and are usually a manifestation of direct physical injury or bone-related diseases such as osteoporosis (Burge et al., 2007). With gradual improvements in healthcare and living, an increase in these cases is predictable because of the longer life expectancies (Rommens, 2019). Although the bone is seen as a rigid structure, it is classified as a connective tissue that constantly remodels itself throughout a human’s lifespan in a highly vascularized environment (Boskey and Coleman, 2010; Cowan and Kahai, 2018). Despite having modest regenerative potential, especially in children, a loss of blood supply in the bone can impede cellular repair and potentially lead to osteonecrosis (Shah et al., 2015). This cut-off in blood circulation can be due to fractures or dislocations that physically cause an interruption. This pathophysiology is commonly observed in the neck of femur (Shah et al., 2015), scaphoid (Hayat and Varacallo, 2018), and talus bone (Matthews and Stitson, 2018).

**FIGURE 2:**
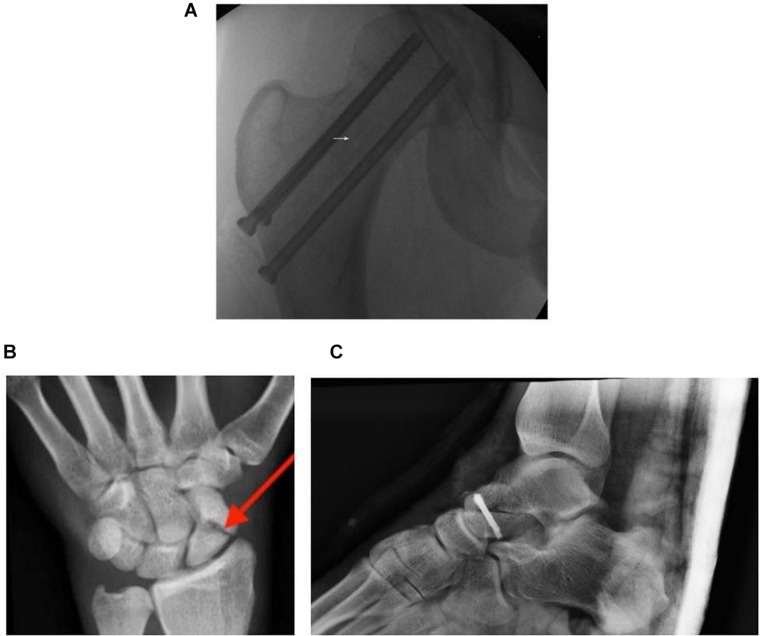
**(A)** An anteroposterior fluorograph of a displaced femoral neck fracture fixed with cannulated screws. The femoral neck (region around white arrow) connects the femoral head to the rest of the femur. **(B)** Plain radiograph of the left anteroposterior carpals of a young adult presented with a full thickness scaphoid fracture (red arrow). **(C)** Post-operative plain radiograph of a talus presented with a fracture. Adapted from Patel (2012), Li et al. (2013), and Kim and Yanuck (2018).

The femur is a piece of bone in the upper leg that articulates with the acetabulum of the pelvic bone and the tibia of the lower leg. A fracture of the femur neck, which connects the femur head to the shaft, has a high risk of cutting off the blood supply by damaging the ascending arteries and causing necrosis (Gumustas et al., 2018). This avascularity in the femur head was found to arise in about 72% of patients who suffered from femur neck fractures, and the incidence increases with severity (Han et al., 2019). One example is a displaced femoral neck fracture (Figure 2A). Fractures of the scaphoid (Figure 2B) are common in the carpals, and are often observed in young, working individuals (Hayat and Varacallo, 2018). Similar to the femur neck, blood vessel interruption in this wrist bone can lead to avascularity and even arthritis if treatment is inadequate (Dias et al., 2016).

Much like how the carpal bones are important for wrist movement, the talus (Figure 2C) is a bone in the lower foot that is crucial for movement. Due to its unique retrograde blood supply, as well as having no direct attachments to any other bones, physical trauma such as from a heavy fall can displace the talus and disrupt vascular supply (Clare and Maloney, 2019). This was found to have a high association with the occurrence of avascular necrosis (Lindvall et al., 2004).

## Hypoxia Limits Orthopedic Treatment Efficiency

Clinical treatment in these cases depends on the severity of the fracture and typically involves an immediate fixation or application of a cast to promote bone union (Matthews and Stitson, 2018). However, the risk of osteonecrosis remains. A more severe injury in the talus, for example, gives rise to more complications regardless of the timing of treatment (Vallier et al., 2004). Even with surgical or non-surgical treatments, bone union and cessation of osteonecrosis are not guaranteed, including the aforementioned bones (Sanders et al., 2004; Dias and Singh, 2011; Gumustas et al., 2018). In recent years, mesenchymal stem cell (MSC) therapy has been used to aid in the regeneration of bone afflicted with osteonecrosis, such as in the femur head (Wang et al., 2019). Since MSCs are multipotent, these stem cells are able to differentiate into osteocytes as the cells are derived from the mesoderm (Fafián-Labora et al., 2019). However, the cut-off in blood supply due to bone fractures can lead to hypoxia-induced changes in engrafted MSCs. Hypoxia is known to affect the proliferation, differentiation, metabolism, and viability of MSCs *in vitro* (Ejtehadifar et al., 2015). Exposure to hypoxia leads to increased cell death, but over time there is an upregulation of stemness genes such as *Oct-4*, *Sox2*, and *Nanog*, as the cells persist in an undifferentiated state (Samal et al., 2021). Interestingly, high oxygen tensions also affect MSCs by reducing proliferative capacity (Krinner et al., 2009).

Besides lowering cell viability, hypoxia has been shown to inhibit metabolic switch and osteogenesis of MSCs (Hsu et al., 2013). The inhibitory effects were demonstrated by suppressed osteogenic markers expression and mineralization of MSCs during osteogenic differentiation (He et al., 2010). Kim et al. (2016) has reported downregulation of calcification, and osteonectin and osteopontin gene expression, implying suppression of osteogenesis in adipose-derived stem cells (ADSCs). It was described that the suppression of osteogenic differentiation involves the generation of reactive oxygen species under oxygen deficiency, which activates Mitogen-Activated Protein Kinase (*MAPK*) and Phosphatidylinositol 3-Kinase/Akt (*PI3K/Akt*) signaling pathways and upregulates intracellular Insulin-like Growth Factor-Binding Protein 3 (IGFBP3) level. Osteogenic differentiation in bone marrow-derived mesenchymal stem cells (BMSCs) was also suppressed under low oxygen condition. Hypoxia of 2% oxygen decreased mineralization and alkaline phosphatase (ALP) activity during osteogenesis (Huang et al., 2012). As such, hypoxia results in a reduction of therapeutic efficacy for bone regeneration due to reduced osteogenesis. Oxygen supplementation can therefore reverse this, and promote bone regeneration by introducing normoxic conditions for osteogenic MSCs. However, this requires careful optimization as too much oxygen supply can lead to hyperoxia and subsequently cell death due to the presence of reactive oxygen species, e.g., hydroxyl radicals (OH-) (Northup and Cassidy, 2008).

Ischemia represents a critical issue during engraftment procedure, especially in the regions without intact vasculature or vascular supply, such as femur, talus, and scaphoid. Persistent ischemia can cause cell death and tissue necrosis (Blaisdell, 2002). Supplementation of oxygen to hypoxic tissue lack of vascularization can ameliorate hypoxia-induced cell death by improving cell rescue and viability. The provision of oxygen is beneficial for tissue regeneration. Supplemental oxygenation of the engineered tissue scaffold can eliminate the hindrance of insufficient oxygen in hypoxic tissues, thus reducing dying or dysfunctional cells in the tissue-engineered graft. Continuous and localized oxygen supply can also delay the onset of necrosis in tissue experiencing hypoxia, resulting in extended tissue viability and improved wound repair. However, the supply of oxygen to hypoxic tissue during engineered tissue engraftment remains the main medical challenge. In the effort to avert ischemia-related cells death, numerous approaches have been studied for the delivery of oxygen to various tissues under hypoxia or ischemia. Creating arteriovenous loops (AV) is one method. In a rat AV loop model, an increase in vascularization was correlated with the increase in Hypoxia Inducible Factor-1 alpha (HIF-1α) rate (Yuan et al., 2018). Another method is to pre-vascularized grafts before transplantation. In a rabbit model, pre-vascularized synthetic bone grafts lead to neovascularization and enhanced bone regeneration (Vidal et al., 2020). Apart from these, the use of scaffolds with oxygen-releasing biomaterials is particularly promising. These can be produced by incorporating the compounds on a 2D scaffold surface or as a 3D capsule. Examples include solid peroxides, liquid peroxides, and fluorinated compounds that function to provide oxygen to cells (Suvarnapathaki et al., 2019).

## Oxygen Releasing Biomaterials

Researchers have designed several oxygen-generating biomaterials and yielded varying degrees of success in enhancing tissue salvage and preserving cellular metabolism under ischemia. Delivering a sustained source of oxygen would represent a major therapeutic advancement in tissue restoration and regeneration following acute trauma and bone defect. Harrison et al. (2007) have reported sustained release of oxygen by Poly(D,L-lactide-*co*-glycolic acid) (PLGA) films integrated with sodium percarbonate (SPO). The implantable oxygen-rich compound SPO can delay tissue death in the hypoxic milieu. The SPO contains sodium bicarbonate and hydrogen peroxide, which readily convert to oxygen upon contact with water as shown in the following chemical equation.

$${\left[{\text{Na}}_{2}\,{\text{CO}}_{3}\right]}_{2}•3\,H_{2}\,{\text{O}}_{2}\to 2\,{\mathrm{Na}}^{+}+2\,C\,O_{3}^{-2}+3\,H_{2}\,{\text{O}}_{2}$$

$$2\,H_{2}\,{\text{O}}_{2}\to O_{2}+2\,H_{2}\,\text{O}$$

The films were also shown to prolong skin cells survival demonstrated by delayed cells degradation, including apoptosis, lactate accumulation, and skin discoloration. The *in situ* production of oxygen has shown to reduce cellular apoptosis and tissue necrosis in the ischemic tissue of mouse model. The effect of cell ischemia continues once the oxygen production is exhausted. This technology employed as a skin wound healing agent was able to delay the onset of necrosis up to 3 days (Harrison et al., 2007). The use of oxygen-releasing peroxide on PLGA films for enhanced cell viability was also validated on a hypoxic fibroblast proliferation study in a 3D tissue-engineered constructs (Oh et al., 2009). Researchers also developed an injectable composite system of PLGA encapsulated in calcium peroxide (CaO_2_)/manganese dioxide (MnO_2_) microparticles (Hsieh et al., 2020). Under low oxygen tension-induced cultures, they were able to promote the differentiation of pre-osteoblast cells. The enhanced local oxygenation by this composite system was found to possess improved bone regeneration potential.

Hypoxic conditions can cause interruption to skeletal muscle metabolism and function. Biomaterials supplementation of oxygen by active element SPO has been demonstrated to support resting skeletal muscle homeostasis under ischemia (Ward et al., 2013). In the study, oxygen-generating SPO mitigated elevations of muscle cells resting tension following contractile fatigue under normoxic conditions. Under oxygen deficiency, SPO lessens HIF-1α build-up, oxidative stress, and ameliorated intramuscular glycogen depletion. SPO administered into ischemia rat skeletal muscle enhanced *in vivo* contractility and ameliorated intramuscular glycogen depletion (Ward et al., 2013). The results indicated that SPO can maintain the contractility of skeletal muscle both *in vitro* and *in vivo* under ischemia. Hypoxia-induced loss of skeletal muscle viability and metabolic homeostasis can also be prevented in the micro-environment with no functioning vasculature.

Calcium peroxide is one important source of oxygen production via its decomposition by water.

$$2\,C\,a\,O_{2}+4\,H_{2}\,\text{O}\to 2\,\mathrm{Ca}\,{\left(\text{OH}\right)}_{2}+2\,H_{2}\,{\text{O}}_{2}\to 2\,\mathrm{Ca}\,{\left(\text{OH}\right)}_{2}+2\,H_{2}\,\text{O}+O_{2}$$

However, the hydrolysis reaction occurs too quickly in the conversion of solid peroxide to oxygen. This causes hyperoxide conditions and leads to the accumulation of reaction intermediate hydrogen peroxide (H_2_O_2_). In addition, the reaction increases the possibility of side reactions such as the production of hydroxyl radical (OH-) (Northup and Cassidy, 2008). Previously reported oxygen releasing biomaterials have mutual limitations, which are less controllable reaction kinetics and short-lived due to the PLGA hydrolytic decomposition. Moreover, cytotoxicity caused by H_2_O_2_ might occur which can lead to cell death as well as reduced ALP activity and mineralization during the osteogenic differentiation process (Lee et al., 2006). Catalase supplementation is always required to counter H_2_O_2_ cytotoxicity by catalyzing its decomposition (Tiedge et al., 1997). Therefore, utilization of such biomaterials can be harmful to transplanted MSCs.

A possible solution for a slower and sustained oxygen release is solid peroxide encapsulation. Calcium peroxide without encapsulation rapidly generates oxygen via hydrolytic conversion, leads to spurts of oxygen that are too transient for cells utilization (Northup and Cassidy, 2008). The bio-stable and hydrophobic (polydimethylsiloxane) PDMS can serve as a diffusional barrier which can decrease the reactivity of the encapsulated calcium peroxide. The capturing of solid peroxide within PDMS were found to regulate oxygen release into the surrounding for over 40 days. Oxygen production by the system is dependent on the water diffusion rate into the PDMS-CaO_2_ disk and the amount of solid peroxide in the disks (Watson and Baron, 1996). PDMS is highly permeable for the oxygen generated to be diffused efficiently out of the system for cellular usage (Robb, 1968). With the controllable features, the concentration of solid peroxide in the disks, and the disks geometry and dimensions can be optimized to design an oxygen generating system with ideal kinetics of oxygen release. The modulation of reactivity of solid peroxide hydrolysis and rapid clearance of end products ensure the dynamic of the forward reaction, thus eliminating the accumulation of hydrogen peroxide intermediate and side reactions such as hydroxyl radicals. As such, PDMS serves as a suitable biomaterial for maintaining viability and function of transplanted cells under ischemic conditions.

Pedraza et al. (2012) have reported the fabrication of a hydrolytically activated oxygen-producing biomaterial. The designed disk of solid calcium peroxide (CaO_2_) encapsulated in hydrophobic polydimethylsiloxane (PDMS), PDMS-CaO_2_ can generate continuous oxygen release for as long as 6 weeks. A single PDMS-CaO_2_ disk was sufficient to regulate cells function of both β cell line and pancreatic rat islets to their normoxic controls. Hypoxia-induced cell dysfunction and death were ameliorated, where the cellular metabolic function and the production of glucose-dependent insulin were regulated as to that under normoxic conditions. Under experimental ischemia, the viability of the β cell line was improved for almost a month with the sustained supply of oxygen from the PDMS-CaO_2_ disk (Pedraza et al., 2012). Ischemia-induced cells apoptosis and dysfunction were prevented by suppression of cell stress pathways activation and shifting to anaerobic metabolism (Lee et al., 2006). Additionally, scaffolds have been used with CaO_2_ to better modulate oxygen release (Touri et al., 2020). These CaO_2_-coated scaffolds were found to augment bone formation at the regions between the scaffolds and the host’s bone. Osteogenic markers such as osteonectin and osteocalcin were upregulated compared to the bone treated with uncoated scaffolds. Other researchers have also developed methods to directly release molecular oxygen instead of hydrogen peroxide-based methods that rely on decomposition to generate oxygen. One such team was able to create hypoxia-sensitive, oxygen molecule-releasing, microspheres and co-injected them with MSCs into mouse ischemic limbs (Guan et al., 2021). They found significant MSCs survival, proliferation, and angiogenesis without the induction of inflammation.

Another approach was conducted to encapsulate pure H_2_O_2_ into a dual-layer matrix to produce clean oxygen for tissue salvage applications (Abdi et al., 2013). The direct usage of encapsulated H_2_O_2_ can prevent the generation of by-products including metal cations, and only yield oxygen and water which are non-toxic (Fatokun et al., 2006). Encapsulated raw H_2_O_2_ normally diffuses out of the biological matrix at a slow pace, increases its direct contact with the cells and the possibility of exerting cytotoxic effect (Abdi et al., 2011). Therefore, the PLGA matrix was coated with catalase-grafted alginate in the study to serve as a protective layer and promote H_2_O_2_ decomposition. In this method, oxygen release can be controlled by manipulating alginate concentration in the microspheres encapsulating H_2_O_2_. Sustained oxygen supply from decomposition of encapsulated H_2_O_2_ in the fabricated dual layered system has shown to enhance cell survival under ischemia (Abdi et al., 2011). The study also revealed optimum H_2_O_2_ concentration to generate efficient amount of oxygen to maintain muscle cells viability under controlled release manner (Abdi et al., 2013). Some researchers have produced a bile acid-based dual-functional prodrug nanoparticle that can scavenge H_2_O_2_, promote osteogenesis, and inhibit adipogenesis of MSCs in a bone defect rat model (Arai et al., 2020). They found a significant improvement in bone regeneration as well as potent anti-inflammatory activities in the MSCs. Other than that, catalase enzymes could be grafted onto microspheres such as poly (L-lactic acid) (PLLA) to speed up the conversion of H_2_O_2_ (Mohseni-Vadeghani et al., 2021). Additionally, by using PLLA to load CaO_2_ and allowing mesenchymal stem cells to adhere to the surface, the oxygen release profile of this system was found to be further sustained. This led to the thought of a potential microcarrier with an injectable cell system for improved bone regeneration.

## Conclusion

Engineered graft implants face major challenges of tissue necrosis and cellular apoptosis upon contact with the hypoxic micro-environment. Provision of sufficient oxygen to the surgery site is of utmost importance for maximum survival and integration of transplanted cells to the wound. Technology to generate oxygen releasing biomaterials as transplantable graft can provide a sustained infusion of oxygen to the local tissue to accelerate tissue regeneration, thereby represents a viable solution for rescuing hypoxic cells. There are, however, potential limitations to this method. A compromised vasculature in the bone defect that persists over the long term could limit the effectiveness of the oxygen-releasing biomaterial since it is transient. Revascularization as a more general treatment against osteonecrosis may yield better long-term recovery, but this requires in-depth studies. Not only that, the failure of mesenchymal stem cell survival after implantation may be due to glucose shortage rather than hypoxia (Moya et al., 2018). As such, exploring glucose-releasing biomaterials could yield surprising results. Still, the importance of vascularization in transplantation and bone repair should still be investigated.

The concept of supplemental oxygen by biomaterials is particularly attractive in orthopedics tissue salvage application. The early bone graft transplantation stages in femur, talus, and scaphoid are susceptible to failure due to the absence of sufficient oxygenation and vascular infiltration. The introduction of engineered tissue graft further aggravates ischemia due to higher metabolic requirements. Sustained *in situ* production of supplemental oxygen appears to be promising for tissue repair applications as tissue graft can remain viable during surgery and proliferate as normal in the hypoxic milieu. The biomaterials help to prevent the development of harmful oxygen gradients that might occur in tissue-engineered implants. Furthermore, oxygenation by biomaterials omits the need for multiple operations to refill engineered tissue grafts due to hypoxic-induced cell loss. Therefore, the provision of supplementary oxygen would serve to enhance cellular viability and benefit the wound healing process. Thus, oxygen-producing biomaterials represent an ideal tool for mitigating oxygenation of orthopedics engineered tissue transplant, specifically for femur, talus, and scaphoid.

## Author Contributions

SWT and AE-HK composed the manuscript and prepared the figures. JBT, XW, AVS, SR, PLM, and SKS commented on the manuscript. SKS and PLM approved the manuscript. All authors reviewed the manuscript.

## Conflict of Interest

The authors declare that the research was conducted in the absence of any commercial or financial relationships that could be construed as a potential conflict of interest.

## Publisher’s Note

All claims expressed in this article are solely those of the authors and do not necessarily represent those of their affiliated organizations, or those of the publisher, the editors and the reviewers. Any product that may be evaluated in this article, or claim that may be made by its manufacturer, is not guaranteed or endorsed by the publisher.
